# Pharmacological Evaluation of Melanocortin 2 Receptor Accessory Protein 2 on Axolotl Neural Melanocortin Signaling

**DOI:** 10.3389/fendo.2022.820896

**Published:** 2022-02-17

**Authors:** Xiaozhu Wang, Song Xue, Xiaowei Lei, Wenqi Song, Lei Li, Xuan Li, Yanbin Fu, Cong Zhang, Hailin Zhang, Yao Luo, Meng Wang, Gufa Lin, Chao Zhang, Jing Guo

**Affiliations:** ^1^ Translational Medical Center for Stem Cell Therapy and Institute for Regenerative Medicine, Shanghai East Hospital, Shanghai Key Laboratory of Signaling and Disease Research, School of Life Sciences and Technology, Tongji University, Shanghai, China; ^2^ Department of Plastic and Reconstructive Surgery, Shanghai Institute of Precision Medicine, Shanghai Ninth People’s Hospital, Shanghai Jiao Tong University School of Medicine, Shanghai, China; ^3^ Key Laboratory of Spine and Spinal Cord Injury Repair and Regeneration of Ministry of Education, Orthopaedic Department of Tongji Hospital, School of Life Sciences and Technology, Tongji University, Shanghai, China

**Keywords:** *Ambystoma mexicanum*, MRAP2, MC3R, MC4R, metabolism

## Abstract

The Melanocortin-3 receptor (MC3R) and Melanocortin-4 receptor (MC4R), two members of the key hypothalamic neuropeptide signaling, function as complex mediators to control the central appetitive and energy homeostasis. The melanocortin 2 receptor accessory protein 2 (MRAP2) is well-known for its modulation on the trafficking and signaling of MC3R and MC4R in mammals. In this study, we cloned and elucidated the pharmacological profiles of MRAP2 on the regulation of central melanocortin signaling in a relatively primitive poikilotherm amphibian species, the Mexican axolotl (*Ambystoma mexicanum*). Our results showed the higher conservation of axolotl *mc3r* and *mc4r* across species than *mrap2*, especially the transmembrane regions in these proteins. Phylogenetic analysis indicated that the axolotl MC3R/MC4R clustered closer to their counterparts in the clawed frog, whereas MRAP2 fell in between the reptile and amphibian clade. We also identified a clear co-expression of *mc3r*, *mc4r*, and *mrap2* along with *pomc* and *agrp* in the axolotl brain tissue. In the presence of MRAP2, the pharmacological stimulation of MC3R by α-MSH or ACTH significantly decreased. MRAP2 significantly decreased the cell surface expression of MC4R in a dose dependent manner. The co-localization and formation of the functional complex of axolotl MC3R/MC4R and MRAP2 on the plasma membrane were further confirmed *in vitro*. Dramatic changes of the expression levels of *mc3r*, *mrap2*, *pomc*, and *agrp* in the fasting axolotl hypothalamus indicated their critical roles in the metabolic regulation of feeding behavior and energy homeostasis in the poikilotherm aquatic amphibian.

## Introduction

As a key neuropeptide hormone family, the melanocortins and their receptors have been reported to participate in numerous physiological functions such as energy homeostasis, natriuresis, inflammation, and exocrine secretion (1, 2). The melanocortins consist of four members, namely, α-, β- and γ-melanocyte-stimulating hormone (MSH) and adrenocorticotropin (ACTH). All of these melanocortins were derived from the polypeptide precursor preprohormone proopiomelanocortin (POMC) through post-translational cleavage in a tissue-specific manner (3, 4). Given the fact that all melanocortins are derived from the same precursor, a tetrapeptide sequence (His-Phe-Arg-Trp) was conserved among them, which involves the melanogenic effects of melanocortins (2). POMC is enriched in the hypothalamus, specifically the arcuate nucleus, where it is processed to ACTH and α-MSH (5–7). To sustain the biological function of the melanocortins, they need to bind with the melanocortin receptors (MCRs), which belong to the seven-pass transmembrane guanine nucleotide-protein coupled receptor (GPCR) family (8).

Five melanocortin receptor subtypes (MC1R–MC5R) have been identified to be distributed in different tissues with segregated biological functions (2, 8, 9). The first MCR family member (MC1R) was cloned in 1992 (10, 11). As a key control element for melanogenesis, MC1R had an abundant expression in melanocytes and melanoma cells (12–15). MC2R, first cloned in the adrenal gland in 1993 (16), could extensively regulate the development, steroidogenesis, glucocorticoid synthesis, and neonatal gluconeogenesis of adrenal gland (17). Shortly after the identification of MC2R, MC3R (18) and MC4R (19) were cloned in the same year. Both MC3R and MC4R were critical for central control of appetite and energy homeostasis, which could be targeted as drug candidates for obesity or anorexia (20–22). MC3R, but not MC4R, was reported to highly express in dopaminergic neurons of the ventral tegmental area (VTA) and regulate the mesolimbic dopaminergic system and reward in a sex-dimorphism manner (23). MC4R widely expressed in the central nervous system (CNS), and its mutations could lead to monogenic obesity in human (24–26). The last MCR family member to be identified was MC5R in 1994 (27). MC5R generally expressed in multiple tissues, involving in exocrine secretion (28), immunoregulation (29) and other physiological functions (30).

In addition to the four melanocortins (α-, β- and γ-MSH and ACTH) and five MCRs (MC1R–MC5R), the melanocortin system also includes two endogenous antagonists of the MCRs: agouti (ASP) (31) and agouti-related protein (AgRP) (32). The binding affinities of five MCRs to their agonists (α-, β- and γ-MSH and ACTH) and antagonists (ASP and AGRP) vary from each other. Human MC1R has the highest binding affinity with α-MSH, almost equivalent affinity with ACTH, but less affinity with β- and γ-MSH (33). MC2R is the ACTH receptor, as ACTH can upregulate MC2R expression and increase the receptor numbers (34). There is no difference in the binding affinity of MC3R for any of the melanocortins, even γ-MSH can activate MC3R at the same extent (35). MC4R has a higher affinity for α-MSH and ACTH, a lower affinity for β-MSH, and the lowest affinity for γ-MSH (2). Surprisingly, MC4R can be antagonized by both ASP and AgRP, which distincts it from MC3R (2). The binding affinity of MC5R with α-MSH and ACTH is higher than β- and γ-MSH (2, 31).

A pair of GPCR single-transmembrane accessory proteins: melanocortin 2 receptor accessory protein (MRAP) and melanocortin 2 receptor accessory protein 2 (MRAP2) also function in the complex melanocortin regulatory system (36). MRAP2 can homodimerize and heterodimerize with MRAP in CHO cells (37). As a specific molecular chaperone for MC2R, MRAP was first reported to actively be involved in the trafficking of MC2R from the endoplasmic reticulum (ER) to the cell surface, suggesting its role as an accessory protein for MC2R (38). Subsequent study further extended the interaction of MRAP and MRAP2 from MC2R to each of the MCRs by Co-IP assay (37). The interaction of MC3R and MC4R with MRAP2 can also alter the MC3R/MC4R cell surface expression. For instance, the co-expression of human MC4R with MRAP or MRAP2 can reduce its cell surface expression, but the cell surface expression of human MC3R was found not to be altered (37, 39).

Several studies have been conducted to evaluate the pharmacological profiles of MC3R/MC4R in the presence of MRAPs in various vertebrates, such as human (40, 41), mouse (42), chicken (43), the clawed frog (44–46), zebrafish (47, 48), snakehead (49), orange-spotted grouper (50), channel catfish (51), topmouth culter (52, 53), and sea lamprey (54). For instance, human MRAP2 was found to promote the maximal activity of MC3R/MC4R stimulated by α-MSH, whereas the mutated MRAP2 variants failed to show the similar effect (40). Moreover, consistent observations were reported for human, chicken, and zebrafish that interaction with MRAP2 made MC4R becomes an ACTH receptor (41, 47, 55).

The melanocortin system, especially MC3R, MC4R, their POMC-derived agonists and endogenous regulator MRAPs, represents a fundamental component to regulate the food intake and energy balance. Multiple studies identified significant increases of adiposity in *Agrp* transgenic or *Pomc*, *Mc3r*/*Mc4r* deficient rodents, suggesting their essential role in body weight regulation and energy metabolism (56–60). For instance, *Mc3r*-deficient mice reveal a normal appetite but increased adiposity (61), whereas *Mc4r*-deficient mice was observed to have an increase in food intake, body weight, body length, and adiposity (62). However, almost all studies regarding melanocortin signaling are conducted in rodents or humans. Only few studies have been conducted to examine the melanocortin signaling in other vertebrates. For instance, MRAPs were identified to improve the MC4R ligand sensitivity to ACTH in chicken (43). Similarly, MRAPs were reported to mediate the pharmacological activities of the neuronal MC3R and MC4R signaling in clawed frog (44–46) and sea lamprey (54). Therefore, the interaction and regulation of the melanocortin signaling in other vertebrates remain unclear and intriguing.

The Mexican axolotl (*Ambystoma mexicanum*) serves as a fascinating tetrapod model for the developmental, evolutionary, and regeneration studies. One of the striking features for the axolotl is its enormous genome (~32 Gb), which consists of 14 chromosomes with large number of repetitive elements. The high percentage of repetitive sequences in the axolotl genome makes the genome assembly and gene annotation extremely difficult. Therefore, tremendous efforts have been made to construct a high-quality reference genome for the axolotl (63–65). The available reference genome allows us to conduct a comprehensive evaluation of the melanocortin system in the axolotl. In this study, we first identified the full-length sequence of *mc3r*, *mc4r*, and *mrap2* in axolotl genome and examined the homology of these genes among species by phylogenetic and synteny analysis. We also revealed the tissue distribution of these genes in adult axolotl and their expression alterations in the hypothalamus after 3-week fasting, and finally elucidated a comprehensively pharmacological evaluation of MRAP2 on melanocortin signaling in axolotl model.

## Methods and Materials

### Animal Protocol Approval Statement

Animal procedures performed in this study were in accordance with the Association for Assessment and Accreditation of Laboratory Animal Care International (http://www.aaalac.org/index.cfm), and the protocols were approved by the Institutional Animal Care and Use Committees (IACUC) of Tongji University.

### Reagents, Plasmids, Antibodies and Primers

All the fragments were ligated into pcDNA3.1(+) plasmid, and the constructed plasmids were then verified by DNA sequencing. The pCre-luc (Santa Cruz, CA, USA) was kindly gifted by Xin Xie’s Lab of Tongji University. Human α-melanocyte stimulating hormone (α-MSH) and human adrenocorticotropin ACTH (1–24) was synthesized by Genescript (Nanjing, China). We purchased the paraformaldehyde (4%), phosphate buffered saline (PBS), bovine serum albumin, non-fat milk powder and β-mercaptoethanol from Sangon Biotech (Shanghai, China). TRNzol Universal Reagent and FastQuant RT Kit (with gDNase) were obtained from Tiangen Biotech (Beijing, China). Tetramethylbenzidine (TMB) chromogen solution was purchased from Beyotime^®^ Biotechnology (Shanghai, China). Hydrochloric acid and sulfuric acid were obtained from Sinopharm Chemical Reagent Co., Ltd (Beijing, China). The antibodies used in this study included Rabbit anti-Flag (Cell Signaling Technology, USA), Mouse anti-HA (Sigma Aldrich, MO, USA), Mouse anti-Flag (Abcam, Cambridge, UK), Goat anti-Mouse IgG (horseradish peroxidase (HRP)-conjugated) (ABclonal Biotech Co., Ltd, Wuhan, China), and Goat Anti-Rabbit Alexa-Fluor 594 (Abcam). All primers used for full-length gene amplification, reverse transcription PCR (RT-PCR), and real-time quantitative PCR are listed in **Supplemental Table 1** and synthesized by GENEWIZ (Suzhou, China).

### Identification of Full-Length of Axolotl *mc3r*, *mc4r*, and *mrap2*

To identify the axolotl *mc3r*, *mc4r*, and *mrap2* gene sequences, we searched the axolotl RNA-Seq dataset with amino acid sequences of the corresponding protein sequences from human (*Homo sapiens*), mouse (*Mus musculus*), cattle (*Bos taurus*), chicken (*Gallus gallus*), common lizard (*Zootoca vivipara*), painted turtle (*Chrysemys picta bellii*), clawed frog (*Xenopus tropicalis*), and zebrafish (*Danio rerio*) as queries using TBLASTN. We then eliminated the duplicates from the initial sequence pool by using Clustal Omega (http://www.ebi.ac.uk/Tools/msa/clustalo/). The resulting unique set of sequences was used for primer design to clone the full-length sequences of these genes. The *mc3r* and *mc4r* were amplified from genomic DNA extracted from axolotl hypothalamus. The *mrap2* was amplified from cDNA of the axolotl brain tissue. The functional domain characteristics and transmembrane helices of *mc3r*, *mc4r*, and *mrap2* were predicted by NCBI Conserved Domain Search (http://www.ncbi.nlm.nih.gov/Structure/cdd/wrpsb.cgi) and TMHMM 2.0 (http://www.cbs.dtu.dk/services/TMHMM/).

### Phylogenetic and Synteny Analysis

The protein sequences of MC3Rs, MC4Rs, and MRAP2s of various different species, namely, Human, Macaque, Sheep, Cattle, Mouse, Chicken, Snake, Common lizard, Painted turtle, Clawed Frog, Rainbow trout, Zebrafish, and Elephant shark were obtained from ENSEMBL genome browser (http://www.ensembl.org/) or NCBI database (http://www.ncbi.nlm.nih.gov/). The details of all used species and sequence information are listed in **Supplemental Table 2**. The multiple alignments of these selected protein sequences were aligned with MAFFT (66). To identify the consensus phylogenetic tree, the best-fit protein evolution model was searched using modeltest NG (67). The final ML phylogenetic tree was inferred with the multiple alignments of amino acid sequences using RAxML v8.2 (68) with the PROTGAMMAJTT protein model (best fit model identified by modeltest NG) and 1,000 rapid bootstrap replicates. Synteny analysis was performed by manually examining adjacent genes of *mc3r*, *mc4r*, and *mrap2* among various species.

### Tissue Distribution Analysis With Reverse Transcription PCR (RT-PCR) and Published RNA-seq Data

Multiple fresh tissues (brain, pituitary, hypothalamus, eye, heart, liver, kidney, spleen, stomach, intestine, ovary, gill, ventral skin, dorsal skin, muscle, fat) from a female adult axolotl were collected, and their total RNA were extracted with TRNzol Universal reagent. The reverse transcription was conducted with 0.5 or 1 μg total RNA from each tissue using FastQuant RT Kit (with gDNase). The expression levels of *mc3r*, *mc4r*, *mrap2*, *agrp*, and *pomc* in each axolotl tissue were determined by RT-PCR, and products were separated on 1.5% agarose gel. To evaluate the expression patterns of *mc3r*, *mc4r*, and *mrap2* in more axolotl tissues, we obtained the public available RNA-Seq data from Axolotl Transcriptomics website (https://portals.broadinstitute.org/axolotlomics/) from a axolotl cross-tissue transcriptome study (69).

### Cell Culture and Transfection

Human Embryonic Kidney HEK293T cells were cultured in Dulbecco’s modified Eagles medium (DMEM) medium with 10% (v/v) fetal bovine serum (FBS) and 1% (v/v) penicillin–streptomycin. Cells were grown in a humidified 5% CO_2_ incubator at 37°C. Transfection was carried out with Polyethyleneimine (PEI) Transfection Reagent (Polysciences, Warrington, PA, USA) according to the manufacturer’s protocol. Empty pcDNA3.1(+) vector were added to maintain the constant total plasmid amounts for all transfections groups.

### cAMP Luminescent Assay

HEK293T cells at a density of 1 × 10^5^ cells/ml were plated into 24-well plates before transfection. The plasmids of axolotl MC3R or MC4R and MRAP2 (at different ratios 1:0, 1:1, 1:3, and 1:6) were transiently co-transfected into HEK293T cells along with constant amounts of pCRE-Luc (firefly luciferase, Santa Cruz, CA, USA) to monitor the cAMP production, and pRL-TK reporter vectors (renilla luciferase, Promega, WI, USA) to normalize the transfection efficiency. Empty vectors were added to each group for maintaining an identical total transfection amount. After 24 h, cell culture medium was replaced with human α-MSH/ACTH (1–24) including indicated concentration in DMEM with 0.1% BSA and incubated for 4 h at 37°C/5% CO_2_, while negative controls were applied by using zero peptide concentration of α-MSH/ACTH (1–24). Luminescent assay was conducted with Dual-Glo Luciferase Assay System (Promega, WI, USA) according to the manufacturer’s protocol. Evaluation of firefly and Renilla luminescence were performed with Spectramax ID3 multimode microplate reader (Molecular Devices, CA, USA). The Firefly luciferase activities of HEK293T cells expressing axolotl MC3R/MC4R were further normalized to Renilla luciferase activities.

### Surface Epitope Detection by Fixed Cell Enzyme Linked Immunosorbent Assay (ELISA)

To examine the effect of axolotl MRAP2 on the cell surface expression of MC3R and MC4R, surface epitope detection by ELISA was conducted in HEK293T cells. In brief, HEK293T cells seeded in a 24-well plate coated with 0.01% poly-D-lysine were transiently transfected with N-terminally 3×HA receptors plasmids with or without different ratios of 2×Flag-MRAP2 plasmids (plasmid ratios of 1:0, 1:1, 1:3, and 1:6). After 24 h transfection, cells were fixed with 4% paraformaldehyde and then washed three times with D-PBS for 5 min each time. Next, cells were incubated for 45 min with blocking buffer (5% milk in D-PBS), followed by incubation with mouse anti-HA antibodies (1:2,000) for 2 h. After incubation, cells were washed again and incubated for 2 h with HRP-conjugated Goat anti Mouse IgG antibodies (1:2,000). Finally, after washing these cells with D-PBS for three times and incubating with TMB for 30 min, the reaction was terminated by sulfuric acid (2 mol/L). We measured the absorbance value at 450 nm using Spectramax ID3 multimode microplate reader.

### Bimolecular Fluorescence Complementation Assay (BiFC)

To detect the interaction between axolotl MC3R/MC4R with MRAP2, HEK293T cells were grown on 0.01% poly-D-lysine coated cover glasses and 2Flag-MC3R/MC4R-VF1 (Venus Fragment 1) and 2Flag-MRAP2-VF2 (Venus Fragment 2) were transfected. 2Flag-MRAP2-VF1 and 2Flag-MRAP2-VF2 were transfected simultaneously as controls. After 24 h of transfection, we removed the culture medium, washed the cells with PBS, and fixed them with 4% paraformaldehyde solution for 15 min at room temperature. Cells washed by PBS were permeabilized, and non-specific binding sites were blocked in PBS containing 5% (w/v) milk for 1 h at room temperature. Permeabilized cells were then incubated overnight with 1:2,000 rabbit anti Flag monoclonal antibodies in PBS with 5% (w/v) milk at room temperature for 2 h and then the cells were washed by PBST (PBS supplement with 0.1% tween-20) 3 times for 5 min each. Then Goat anti-rabbit Alexa-Fluor 594 secondary antibodies (1:1,000) were added and incubated for 2 h in the dark. Finally, the coverslips were mounted with antifade reagent with DAPI (Cell Signaling Technology, USA) on microscope slides. Confocal microscopy imaging was conducted with a ×63 oil objective on a Zeiss confocal microscope, and the micrographs displayed in each group were exposed and identically processed.

### Co-Immunoprecipitation and Western Blot

To explore the protein interaction between the axolotl MC3R/MC4R and MRAP2, the N-terminally Flag tagged MRAP2 (2xFlag-MRAP2) and N-terminally 3×HA tagged receptors expression plasmids were constructed by cloning the coding sequence into the pcDNA3.1(+) vector. The tagged receptors and MRAP2 expression plasmids were then co-transfected into HEK293T cells and incubated for 36 h. The transfected cells were then lysed by lysis buffer with 0.75% TritonX-100 [150 mM NaCl,50 mM Tris–HCl (pH 7.9) and proteinase inhibitor cocktail (Roche, Penzberg, Germany)]. The co-IP experiment was conducted with supernatants centrifuged from the cell lysates by overnight incubating the samples with rabbit anti HA antibody at 4°C. Protein A/G Agarose beads (Beyotime, Shanghai, China) were added to the cell lysates right after the overnight incubation and rotated at 4°C for 3–4 h. Beads were then washed three times in lysis buffer and centrifuged, re-suspended in SDS loading buffer. After boiling for 15 min, proteins separated on sodium dodecyl sulfate-polyacrylamide gel (SDS/PAGE, 12%) electrophoresis along with the mouse anti-HA antibodies for receptor proteins and mouse anti-Flag antibodies for MRAP2 proteins were used to perform western blot.

### Food Deprivation Analysis and Real-Time Quantitative PCR Evaluation

To evaluate the metabolic expression changes of *mc3r*, *mc4r*, *mrap2*, *pomc*, and *agrp* after three-week fasting in the axolotl, 6 juvenile axolotls were used for the food deprivation experiment with 3 as a control group and 3 in the experimental group. All juvenile axolotls from both groups were kept at the room temperature with sufficient oxygen supplements, and water in each tank was changed every day. The animals in the control group were fed twice a day for 3 weeks, whereas the animals in the experimental group were fasted for 3 weeks without any food. The body weight for each axolotl was measured before and post the experiment. The hypothalamic tissues from both control and fasting group were dissected and stored in TRNzol. The total RNA extraction and first-strand reverse transcription were conducted with TRNzol Universal reagent and FastQuant RT Kit (with gDNase) following the standard protocols. We then diluted the cDNA reaction to 1:10 as a template for qPCR. The qRT-PCR was performed using SuperReal PreMix Plus (SYBR Green) with the PCR setting as following: preincubation at 95°C for 600 s; 45 cycles of 95°C 10 s, 60°C for 10 s, and 72°C for 10 s; melting at 95°C for 10 s, 65°C for 60 s and 97°C for 1 s. All reactions were run in three biological replicates for both control and fasting groups and repeated three times.

### Data Analysis

To validate the results of all experiments, we separately repeated these experiments at least three times. The dose–response curves for cAMP luminescent assay were analyzed with the nonlinear regression models, and the evaluation of the corresponding half-maximal effective concentration (EC_50_) values was calculated using GraphPad Prism 6 (GraphPad Software, Inc., CA, USA). The receptor surface expression was also analyzed using GraphPad Prism 6. We applied one-way ANOVA with Tukey post-test with a nominal significant level of 0.05 (*p <0.05). The final results were represented as mean ± SEM.

## Results

### Identification of *mc3r*, *mc4r*, and *mrap2* Genes in Axolotl

Using multiple blast tools from the NCBI database, we identified the full-length gene sequences of the melanocortin receptors (*mc3r*, *mc4r*) and melanocortin accessory protein (*mrap2*) in the axolotl genome (63–65). The axolotl *mc3r* contains 963 coding nucleotides for 320 amino acids and are located in Chr3P; *mc4r* contains 1,008 coding nucleotides for 324 amino acids and are located in Chr5P; and *mrap2* contains 651 coding nucleotides for 216 amino acids and are located in Chr4P. Both axolotl MC3R and MC4R contain seven transmembrane (TM) regions like other GPCRs, while MRAP2 includes one TM domain like MRAPs in other species.

**Figure 1:**
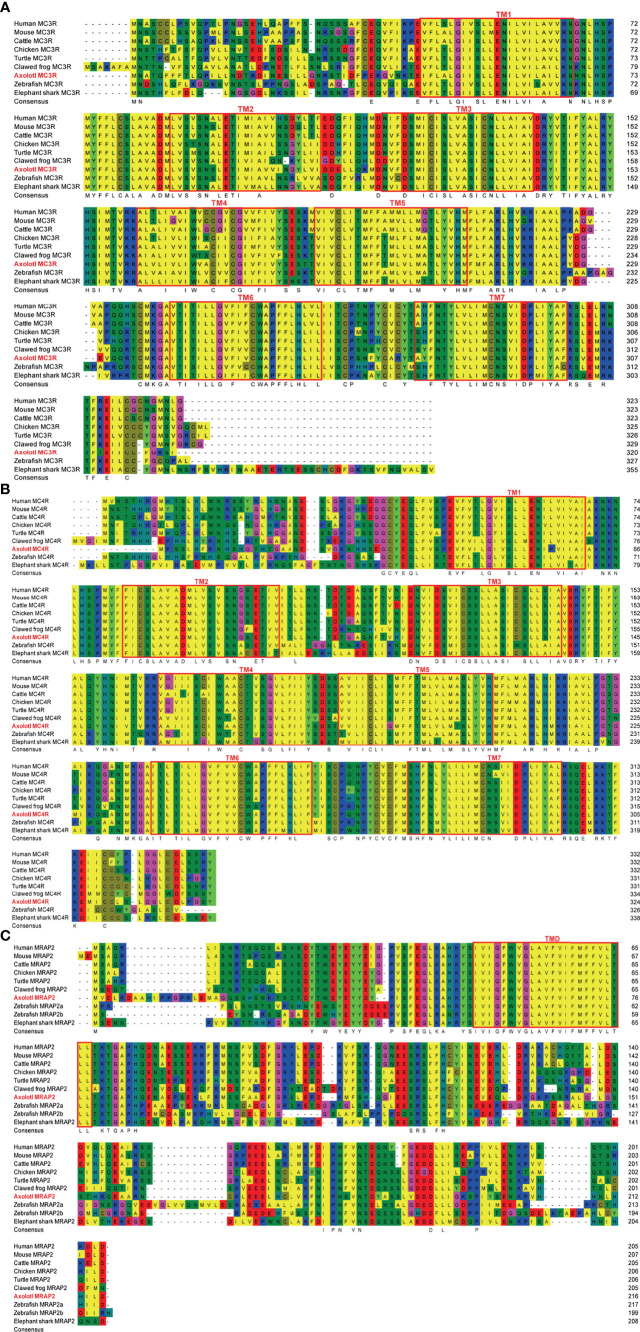
Sequence alignments of multiple MC3R, MC4R, and MRAP2 proteins. **(A)** Sequence alignment of multiple MC3R proteins. **(B)** Sequence alignment of multiple MC4R proteins. **(C)** Sequence alignment of multiple MRAP2 proteins. The transmembrane (TM) regions in the amMC3R and amMC4R are represented by red box and are numbered 1–7. The red box marks the transmembrane domains (TM) in the MRAP2.

The multiple alignments of MC3R, MC4R, and MRAP2 protein sequences were carried out for representative vertebrates selected from each class, namely, primate (Human, Macaque), ruminant (Cattle, Sheep), rodent (Mouse), avian (Chicken), reptile (Turtle, Snake, Common lizard), amphibia (Clawed frog, Axolotl), teleost (Zebrafish, Rainbow trout), and cartilaginous fish (Elephant shark), as shown in **Figures 1A–C**, respectively (**Supplemental Table 2**). The axolotl MC3R and its orthologs across species have an average sequence identity 72%, sharing the highest identity with chicken (80%), and the lowest identity with rainbow trout (66%) (**Figure 1A**). In comparison with the axolotl MC3R, MC4R shares higher sequence identities (average identity of 80%) with its orthologs across species, namely, the lowest identity of 71% with zebrafish and the highest identity of 85% with turtle (**Figure 1B**). Notably, sequences in the 7 TMs for both MC3R and MC4R are more conserved across various species than that in the C-terminal and N-terminal. The axolotl MRAP2 and its orthologs across species has a relatively low sequence identity (average identity of 59%), whereas a striking homology was found in the transmembrane domain, suggesting its importance for maintaining conserved function of MRAP2.

### Phylogenetic and Synteny Analysis of Axolotl MC3R, MC4R and MRAP2

**Figure 2:**
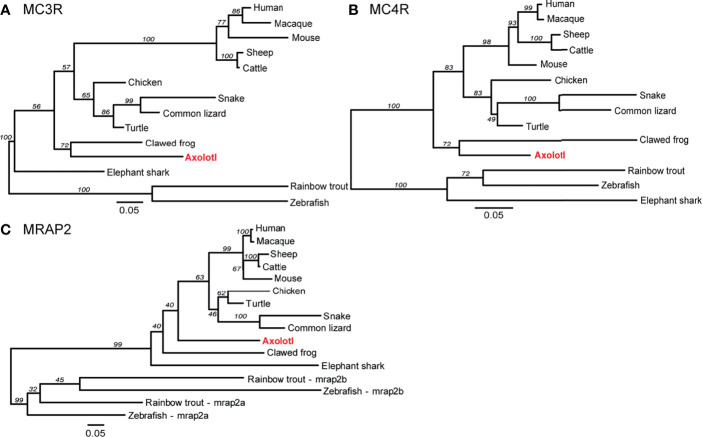
Phylogenetic analysis of MC3R, MC4R, and MRAP2. Phylogenetic trees of **(A)** MC3R, **(B)** MC4R, and **(C)** MRAP2. The ML phylogenetic tree was inferred based on multiple alignments of protein sequences using RAxML v8.2 with the PROTGAMMAJTT protein model and 1,000 rapid bootstrap replicates.

To reveal the phylogenetic relationship of these melanocortin genes, the ML phylogenetic trees were constructed with the protein sequences of the representative vertebrates. The consensus phylogenetic trees of MC3R, MC4R, and MRAP2 are shown in **Figure 2**. The axolotl MC3R was grouped into the amphibian clade, and mostly related to the clawed frog MC3R with a bootstrap value of 72 (**Figure 2A** and **Supplemental Table 2**). Similarly, the axolotl MC4R was grouped into the amphibian clade, and mostly related to the clawed frog MC4R with a bootstrap value of 72 (**Figure 2B** and **Supplemental Table 2**). Unlike the axolotl MC3R/MC4R, MRAP2 was not grouped with the clawed frog MRAP2, but instead the axolotl MRAP2 fell in between the reptile and amphibian clade (**Figure 2C** and **Supplemental Table 2**). The phylogenetic relationships of MC3R and MC4R for other species were generally in accordance with their evolutionary clades, except for the elephant shark MC3R and MRAP2 which were grouped more closely with other non-teleost fish species instead of zebrafish and rainbow trout.

**Figure 3:**
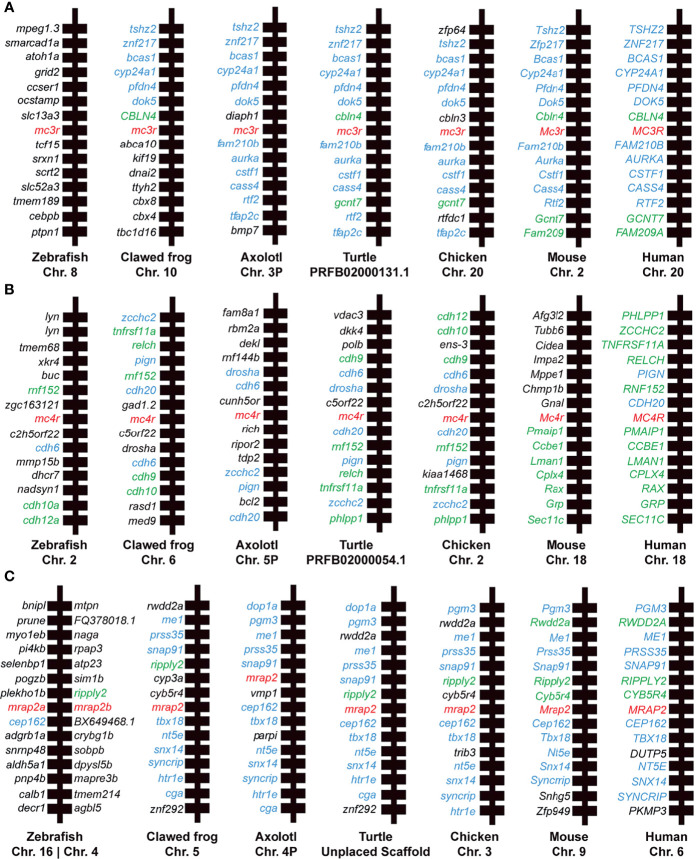
Synteny analysis of *mc3r*, *mc4r*, and *mrap2*. Chromosomal location and adjacent genes of **(A)** *mc3r*, **(B)** *mc4r*, and **(C)** *mrap2* in following species: primate (Human, Macaque), ruminant (Cattle, Sheep), rodent (Mouse), avian (Chicken), reptile (Turtle, Snake, Common lizard), amphibia (Clawed frog, Axolotl), teleost (Zebrafish, Rainbow trout), and cartilaginous fish (Elephant shark). Genes showing conserved synteny with axolotl are shown in blue. Genes showing conserved synteny among at least two species but not with axolotl are shown in green.

Synteny analysis was performed to provide additional aspects for the homology of *mc3r*, *mc4r*, and *mrap2* genes across species, including human, mouse, chicken, turtle, clawed frog, zebrafish, and axolotl. We found that all flanking genes of axolotl MC3R shared flanking genes in other species except for *diaph1* and *bmp7* (as shown in blue, **Figure 3A**). The syntenic blocks of *mc3r* in axolotl were generally conserved with turtle, chicken, mouse, and human, while the flanking genes of *mc3r* in clawed frog and zebrafish varied from other species. In contrast, 5 out of 14 *mc4r* flanking genes in axolotl were conserved with the flanking genes in most species (as shown in blue, **Figure 3B**), suggesting that the syntenic blocks of axolotl *mc4r* were much less conserved with other species than that in axolotl *mc3r*. Moreover, we noticed that the flanking genes in other species, especially human, mouse, chicken, and turtle, were much more conserved with each other than with axolotl (shared flanking genes among other species without axolotl are shown in green, **Figure 3B**). The syntenic blocks of axolotl *mrap2* were generally conserved with other species, especially between axolotl and turtle (**Figure 3C**). Similar with axolotl *mc3r*, 12 out of 14 *mrap2* flanking genes in axolotl shared homologous in other species except for both copies (*mrap2a* and *mrap2b*) in zebrafish. The adjacent genes of two mrap2 copies in zebrafish were hardly overlapped with other species, suggesting a unique evolutionary pattern in teleost.

### Tissue Distribution Analysis of *mc3r*, *mc4r*, *mrap2*, *agrp*, and *pomc* Genes in Axolotl

**Figure 4:**
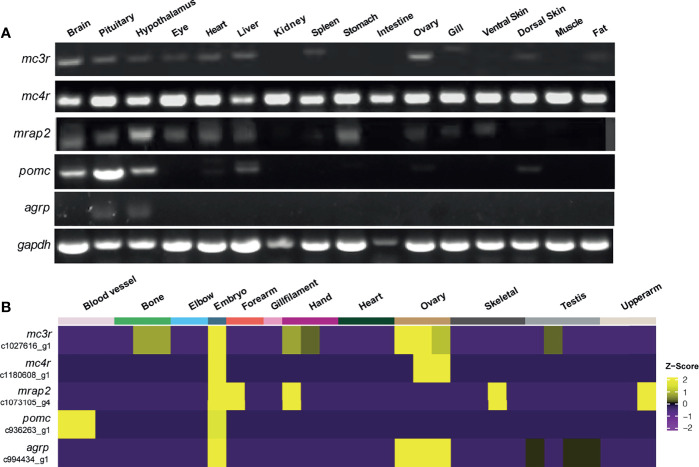
Tissue distribution of *mc3r*, *mc4r*, *mrap2*, *agrp*, and *pomc* mRNA in axolotl. **(A)** Tissue expression of *mc3r*, *mc4r*, *mrap2*, *agrp*, and *pomc* mRNA was analyzed by semi‐quantitative reverse transcription PCR using a housekeeping gene glyceraldehyde 3-phosphate dehydrogenase (*gapdh*) as the internal control. **(B)** Heatmap showing the corresponding transcripts of *mc3r*, *mc4r*, *mrap2*, *agrp*, and *pomc* enriched in specific tissue types.

To identify the tissue distribution patterns of *mc3r*, *mc4r*, *mrap2*, *agrp*, and *pomc* in axolotl, quantitative RT-PCR analysis was performed with the cDNA libraries from 16 tissues of a female adult axolotl (**Figure 4A**). Moderate but clear expression of *mc3r*, *mc4r*, *mrap2*, *agrp*, and *pomc* were detected in the axolotl brain, hypothalamus and pituitary. In addition, axolotl *mc3r* expression was detected in eye, heart, liver, spleen, ovary, dorsal skin and fat, whereas axolotl *mc4r* showed a broad expression pattern across multiple tissues in axolotl. Similar with *mc3r*, axolotl *mrap2* also expressed in several peripheral tissues including eye, heart, liver, stomach, ovary, gill and ventral skin. The axolotl *agrp* and *pomc* showed restricted expression profiles in brain, with very low level of *pomc* in liver, ovary, and dorsal skin.

To reveal the expression profiles in more axolotl tissues, we obtained the published bulk RNA-seq data of axolotl tissues and annotated the corresponding transcripts for *mc3r*, *mc4r*, *mrap2*, *agrp*, and *pomc* to illustrate the expression patterns in various tissues (**Figure 4B**) (69). Interestingly, all 5 genes were highly expressed in axolotl embryo, suggesting their potential functional involvements for the embryonic development.

### Stimulation of MC3R and MC4R by α-MSH and ACTH in the Presence of MRAP2 Protein

**Figure 5:**
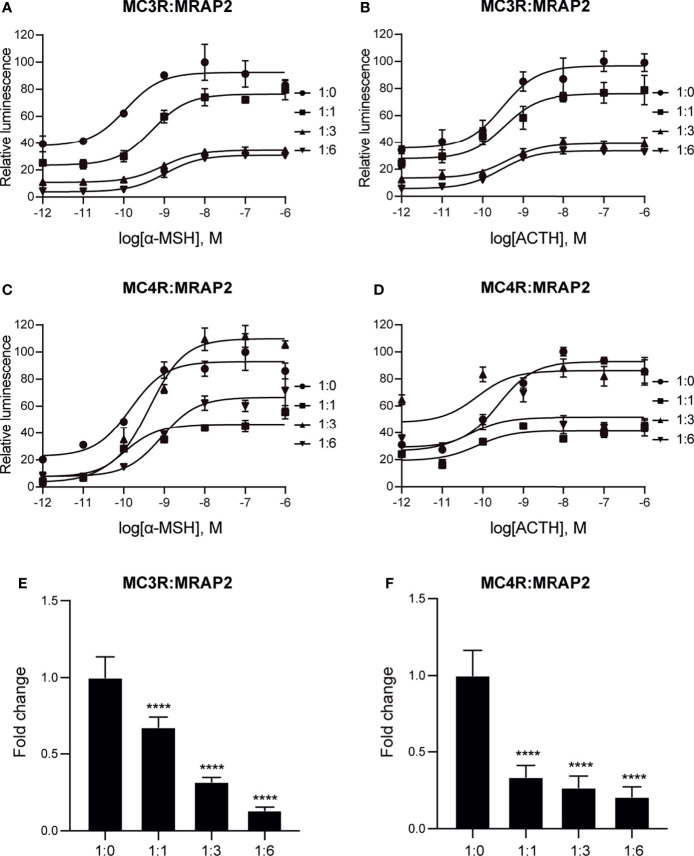
Pharmacological modulation of axolotl MC3R and MC4R by α-MSH and ACTH. **(A)** Effect of different doses (10^−12^–10^−6^ M) of α-MSH in activating axolotl MC3R in the presence of axolotl MRAP2 in HEK293T cells measured with Dual-glo luciferase assay. **(B)** Effect of different doses (10^−12^–10^−6^ M) of ACTH in activating axolotl MC3R in the presence of axolotl MRAP2 in HEK293T cells measured with Dual-glo luciferase assay. **(C)** Effect of different doses (10^−12^–10^−6^ M) of α-MSH in activating axolotl MC4R in the presence of axolotl MRAP2 in HEK293T cells measured with Dual-glo luciferase assay. **(D)** Effect of different doses (10^−12^–10^−6^ M) of ACTH in activating axolotl MC4R in the presence of axolotl MRAP2 in HEK293T cells measured with Dual-glo luciferase assay. **(E, F)** The constitutive activity of MC3R/MC4R in the presence of different amounts of MRAP2. ****p < 0.0001.

Next, we performed cAMP luminescent monitoring assay in HEK293 cells upon treatment by α-MSH or ACTH in the presence of different concentrations of axolotl MRAP2 (1:0, 1:1, 1:3, and 1:6) to characterize the pharmacological profiles of axolotl MC3R and MC4R *in vitro*. As shown in **Figures 5A, B**, we found that the luciferase signal of MC3R could be potentiated by α-MSH and ACTH without MRAP2 (logEC_50_ of α-MSH −9.95 ± 0.35 M and logEC_50_ of ACTH −9.51 ± 0.36 M, **Table 1**). Similar with MC3R, we also verified the stimulation of MC4R by α-MSH with logEC_50_ value of −9.84 ± 0.31 M and ACTH with logEC_50_ value of −9.62 ± 0.30 M in a dose-dependent manner (**Figures 5C, D** and **Table 1**). Strikingly, axolotl MC3R and MC4R both exhibited noticeable constitutive activities in the presence of MRAP2 proteins.

**Table 1:** EC_50_ values of α-MSH and ACTH in activating axolotl MC3R/MC4R in presence of different amounts of axolotl MRAP2.

		1:0	1:1	1:3	1:6
MC3R:MRAP2 (α-MSH)	logEC_50_	−9.95 ± 0.35	−9.29 ± 0.21	−9.04 ± 0.13	−8.98 ± 0.13
	*P-value*		ns	***	****
MC3R:MRAP2 (ACTH)	logEC_50_	−9.51 ± 0.36	−9.47 ± 0.51	−9.40 ± 0.28	−9.55 ± 0.14
	*P-value*		ns	**	***
MC4R:MRAP2 (α-MSH)	logEC_50_	−9.84 ± 0.31	−10.02 ± 0.40	−9.37 ± 0.22	−9.06 ± 0.22
	*P-value*		ns	ns	ns
MC4R:MRAP2 (ACTH)	logEC_50_	−9.62 ± 0.30	−10.13 ± 0.47	−10.14 ± 1.04	−10.33 ± 0.78
	*P-value*		*	ns	ns

Data shown are the mean of ± SEM of three replicates. One-way ANOVA with Tukey post-test were performed to measure significance between the MC3R/MC4R expressed alone group (1:0) and the experimental groups. *p < 0.05, **p < 0.01, ***p < 0.001, ****p < 0.0001, ns, not significant.

To further evaluate how axolotl MRAP2 proteins alter the stimulation of axolotl MC3R and MC4R by α-MSH or ACTH *in vitro*, we examined different transient transfection ratios of MCRs to MRAP2 (1:0, 1:1, 1:3, 1:6). Noticeably, the stimulation of MC3R was dramatically suppressed by MRAP2 (**Figures 5A, B** and **Table 1**). The presence of MRAP2 could dose‐dependently decrease both the constitutive activity and maximal responsive plateau of MC3R by either α-MSH or ACTH (**Figures 5A, B, E** and **Table 1**). Similarly, the co-expression of MRAP2 revealed a mild alteration on the MC4R stimulation by two agonists (**Figures 5C, D** and **Table 1**). With different concentrations of MRAP2, the stimulation of MC4R by α-MSH and ACTH generally showed no significant change (p >0.05). The constitutive activity of MC4R significantly decreased in a dose-dependent manner with various proportions of MC4R and MRAP2 plasmids (**Figure 5F**).

### Modulation of the Cell Surface Expression of MC3R and MC4R by MRAP2 in Axolotl

**Figure 6:**
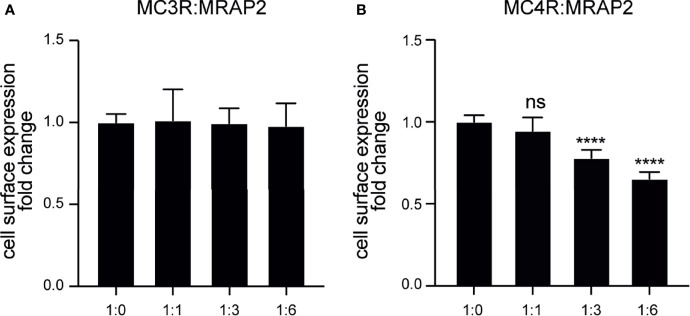
Modulation of the cell surface expression of axolotl MC3R and MC4R by MRAP2. **(A)** Cell surface expression of axolotl MC3R in presence of different amounts of MRAP2 (1:0, 1:1, 1:3, 1:6). **(B)** Cell surface expression of axolotl MC4R in presence of different amounts of MRAP2 (1:0, 1:1, 1:3, 1:6). The cell surface expression level of each receptor was expressed as the fold change of the measured surface epitopes level when the receptor was expressed alone (at the plasmid ratio of 1:0). Each data point represents mean ± SEM of three replicates (N = 3). One-way ANOVA with Tukey post-test were performed for all groups of data (****p < 0.0001, ns, not significant).

To investigate the role of axolotl MRAP2 in the trafficking of axolotl MC3R/MC4R to the cell membrane, we conducted ELISA assay to measure the cell surface expression level of MC3R/MC4R in the presence of various ratios of MRAP2 plasmids. As shown in **Figure 6A**, no significant change was found in the cell surface expression level of MC3R in the presence of any amounts of MRAP2, suggesting that MRAP2 could not significantly affect the trafficking of MC3R to the cell membrane. Unlike MC3R, the cell surface expression of MC4R significantly decreased when co-expressed with MRAP2 in the ratios of 1:3 and 1:6 (**Figures 6B**). Moreover, the presence of MRAP2 decreased MC4R cell surface expression in a dose-dependent manner because the lower amount of MRAP2 (1:1) failed to significantly decrease the cell surface expression level of MC4R (**Figure 6B**). Taken together, the distinct effects of MRAP2 on the cell surface expression of MC3R and MC4R in axolotl suggested a complex mechanism of MRAP2 for modulating trafficking and post-translational cell surface translocation of MCRs.

### The Interaction of Axolotl MRAP2 With MC3R and MC4R Proteins *In Vitro*

**Figure 7:**
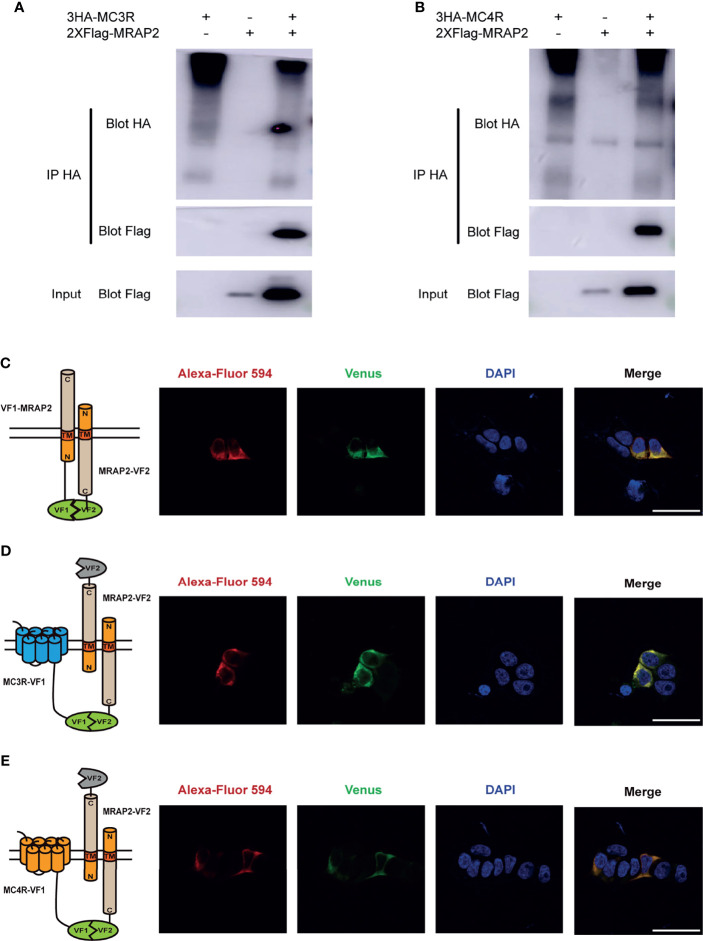
Interaction and co-localization of axolotl MC3R/MC4R and MRAP2 *in vitro*. **(A)** Coimmunoprecipitation of 3HA‐MC3R with 2xFlag‐MRAP2 in HEK293T cells. **(B)** Coimmunoprecipitation of 3HA‐MC4R with 2xFlag‐MRAP2 in HEK293T cells. **(C)** Confocal microscopy of the co-localization of MRAP2-VF1 and MRAP2-VF2 on the plasma membrane. **(D)** Confocal microscopy of the co-localization of MC3R and MRAP2 on the plasma membrane. **(E)** Confocal microscopy of the co-localization of MC4R and MRAP2 on the plasma membrane. Nuclei stained with DAPI are shown in blue and Venus fluorescence in green. Surface expression of MC3R/MC4R, and MRAP2 is shown in red, detected by anti‐Flag antibody and secondary anti‐rabbit Alexa Fluro 594 (Abcam). Scale bar = 50 µm.

Given the fact that axolotl MRAP2 could regulate the trafficking and signaling of MC3R and MC4R, we further monitored the protein complex formation and co-localization *in vitro* by co-IP and BiFC assays in HEK293T cells. We found that both axolotl MC3R and MC4R could interact with MRAP2 and the coimmunoprecitated bands were observed on the SDS-PAGE gel (**Figures 7A, B**). To examine the co-localization of axolotl MC3R/MC4R with MRAP2, we conducted the BiFC assays using two complementary fragments of Venus fluorescent protein: Venus-F1/VF1 fused to MC3R/MC4R and Venus-F2/VF2 fused to MRAP2. The MRAP2 control experiment indicated a clear co-localization of MRAP2 with two different tags (**Figure 7C**). As shown in **Figure 7D**, we detected a stable fluorescence on the plasma membrane of MRAP2 and MC3R, suggesting their co-localization on the cell surface. Similarly, a steadily detectable fluorescence of MRAP2 and MC4R was also identified on the plasma membrane, confirming the co-localization and functional protein complex of MRAP2 and MC4R (**Figure 7E**).

### Dynamic Expression Changes of *mc3r*, *mc4r*, *mrap2*, *agrp*, and *pomc* in Axolotl Hypothalamus Upon 3-Week Food Deprivation

**Figure 8:**
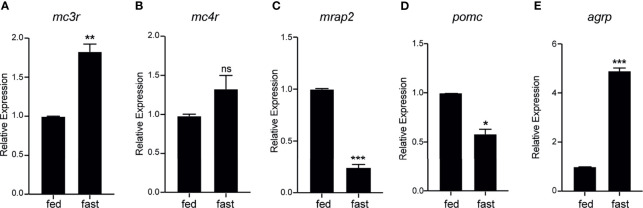
Fasting induced expression dynamics of *mc3r*, *mc4r*, *mrap2*, *agrp*, and *pomc* in the axolotl hypothalamus. Juvenile axolotls were fasted for 3 weeks and the expression levels of **(A)** *mc3r*, **(B)** *mc4r*, **(C)** *mrap2*, **(D)** *pomc*, and **(E)** *agrp* in the hypothalamus were analyzed by qRT-PCR. Data are analyzed by two-tailed t-test compared with the normal feeding group, graphs are shown as mean ± SEM. *P < 0.05, **P < 0.01; ***P < 0.001; ns, not significant.

To evaluate the metabolic expression dynamics of *mc3r*, *mc4r*, *mrap2*, *agrp*, and *pomc* in axolotl hypothalamus, we conducted a 3-week food deprivation experiment, and carried out qRT-PCR to compare the hypothalamic expression levels between the normal feeding and the 3-week fasting group. The axolotl *mc3r* was significantly upregulated in the fasting hypothalamus (**Figure 8A**), while *mc4r* had a mild but not significant upregulation upon fasting (**Figure 8B**). After 3-week fasting, the expression level of *mrap2* significantly was downregulated with a P-value of 0.0007 (**Figure 8C**). Similar to previous fasting studies, *pomc* was significantly downregulated in hypothalamus after fasting, whereas *agrp* revealed an opposite pattern with significantly upregulation in the fasting hypothalamus (**Figures 8D, E**).

## Discussion

The vital physiological roles of MC3R/MC4R and MRAP2 on the regulation of food intake and energy expenditure have been well studied in mammals, especially in human and mouse. As a phylogenetically important tetrapod species, the axolotl shares a similar vertebrate body shape with human but owns a remarkable capability to regenerate limbs and other tissues upon severe damage. We have previously shown that proper activation of MC4R signaling was essential for the limb regeneration in the *Xenopus* model (55). However, the pharmacological profiles of melanocortin receptor and the accessory protein system remain unknown in the axolotl. Here in this study, with multiple experimental approaches, we investigated the expression and function of axolotl neural melanocortin signaling. We cloned the full-length coding sequences of axolotl *mc3r*, *mc4r*, and *mrap2* and performed the phylogenetic and synteny analysis of these genes among multiple species. We also examined the tissue expression patterns of these genes in axolotl and evaluated the pharmacological profiles of axolotl MC3R/MC4R in the presence of MRAP2. Finally, we determined the expressional dynamics of *mc3r*, *mc4r*, *mrap2*, *pomc*, and *agrp* in the fasting axolotl hypothalamus. This is the first comprehensive pharmacological evaluation of MRAP2 on axolotl neural melanocortin signaling.

In this study, obtaining the full-length sequences of axolotl *mc3r*, *mc4r*, and *mrap2* required extra efforts due to the lack of complete gene annotation of the axolotl genome. Similar to other species, axolotl *mc3r* and *mc4r* are also intronless. So we cloned the full-length sequences from the hypothalamus derived genomic DNA. The full length of axolotl MC3R/MC4R protein is 320 and 324 amino acids, respectively, which is similar to the corresponding homology of other species (high identity >65%). The axolotl MRAP2 consists of 216 amino acids but is less conserved comparing to the corresponding genes of other species (average identity of 59%). The phylogenetic analysis indicates that both axolotl MC3R and MC4R are closely grouped with the corresponding counterparts in the clawed frog, suggesting the potential functional similarity of these MCRs among these species. We next compared the flanking genes of *mc3r* and *mc4r* across 7 different species and found the flanking region of *mc3r* was more conserved among these species than that of *mc4r*. Syntenic blocks of *mc3r* and *mc4r* in the zebrafish varied evidently from other species, indicating an early evolutionary separation of these genes in teleosts. The phylogenetic analysis of MRAP2 revealed a less conserved relationship among species, and axolotl MRAP2 fell in between the reptile and amphibian clade. A unique feature of two copies of *mrap2* existent in the zebrafish and rainbow trout genome, suggests that there could be a teleost-specific duplication of *mrap2* genes in the teleosts. The synteny analysis of *mrap2* revealed that the flanking region of *mrap2* was generally conserved among species, except for that in the zebrafish.

The tissue distribution of *mc3r*, *mc4r*, and *mrap2* are identified in many species. In human, *MC3R* is enriched in the brain region and has a very low abundance in other peripheral tissues such as testis and adrenal gland. Human *MC4R* exhibits high expression level in the brain, fallopian tube, and retina, moderate abundance in thalamus, olfactory bulb, and spinal cord, but low abundance in salivary gland, adrenal gland, spleen, pituitary, stomach, testis, and ovary. In addition to high expression level of human brain, *MRAP2* shows a broader distribution among other human tissues than *MC3R*/*MC4R*, indicating that its function might not limit to the energy homeostasis. In the clawed frog, *mc3r* and *mc4r* are expressed in brain region with low abundance in some peripheral tissues, whereas *mrap2* distributes widely among multiple tissues including brain (45, 46). Moreover, in the zebrafish only *mrap2a* expressed in larval zebrafish, while *mrap2b* expression emerged until the adult stage, suggesting that these two genes could participate in the metabolic regulation at different life stages (48). In our study, a clear co-expression of *mc3r*, *mc4r*, *mrap2*, *pomc*, and *agrp* was observed in the axolotl brain region, indicating the potential interaction of these 5 genes for regulating the feeding behavior and energy metabolism in the central nervous system of axolotl.

MRAP2 could interact with MCRs and alter their sensitivity to α-MSH and ACTH in a bi-direction manner among different species. Human MRAP2 was reported to interact with all MCRs (37), and the co-expression of MRAP2 could change MC4R to an ACTH receptor (41). However, mouse MRAP2 could enhance the receptor sensitivity of MC4R to the α-MSH (42). In the clawed frog, MC3R and MC4R could respond to both α-MSH and ACTH, but α-MSH or ACTH seems to be more efficient for activating MC3R than MC4R (45). In chicken, MC3R and MC4R responded to α-MSH and ACTH equivalently, however, MC4R was found to become ACTH-preferred receptor in the presence of MRAP2 (43). Similarly, zebrafish MRAP2a facilitated the pharmacological profile of MC4R by preferably increasing its response to ACTH (47). In tilapia, MRAP2 was reported to reduce the MC4R signaling in response to α-MSH (70), whereas the feline MRAP2 was shown to enhance MC4R signaling stimulated by α-MSH (71). Here, we conducted cAMP luminescent assay to evaluate the pharmacological profiles of axolotl MC3R and MC4R in response to α-MSH and ACTH *in vitro*. We identified a clear stimulation of MC3R and MC4R by α-MSH in a dose-dependent manner (**Figures 5A, B**). These findings strongly suggested a conserved physiological function of axolotl MC3R and MC4R in response to the natural agonist α-MSH.

The constitutive activities of MCRs can affect the regulation of their physiological functions. We found that axolotl MC3R exhibited a high constitutive activity, that was significantly decreased by MRAP2 (**Figure 5E**). MC3R was also reported to have high constitutive activity in other species, such as zebrafish (72), channel catfish (51), and topmouth culter (53). In contrast to our result, the constitutive activity of MC3R was reported to be enhanced by both MRAP and MRAP2 in the clawed frog (45). MC4R exhibits strong constitutive activity in mammals (73–75), chicken (43), and amphibians (44, 45). Moreover, many teleost species have been reported to detect high constitutive activities of MC4R, namely, zebrafish (48), spotted scat (76), grass carp (77), swamp eel (78), Orange-spotted grouper (50), spotted sea bass (79), Nile tilapia (70), topmouth culter (52), and snakehead (49). Several MC4R mutants identified from obese patients have been reported to show a decreased constitutive activity but no other functional changes compared to normal MC4R (80). Constitutive activity defect of MC4R leads to obesity suggesting the basal component activity of MC4R acts as a tonic satiety signal, which may be necessary to maintain long-term energy homeostasis in human. In our study, we demonstrated the native basal cAMP activity of axolotl MC3R/MC4R signaling. MRAP2 reduced the constitutive activities of MC3R and MC4R indicating that MRAP2 could negatively influence the endogenous signaling cascades of axolotls.

Next our ELISA assay demonstrated that axolotl MRAP2 significantly decreased the cell surface expression of MC4R in a dose-dependent manner but did not affected the trafficking of MC3R. In other species, MRAP2 showed varied effect in the modulation of MC3R/MC4R translocation to the cell surface. For instance, the surface expression of clawed frog MC3R was suppressed by MRAP2 (45), whereas in our study, axolotl MRAP2 did not affect the cell surface expression of MC3R. MRAP2 showed no effect on cell surface expression of MC4R in both chicken (43) and snakehead (49). However, MRAP2 could significantly decrease the surface level of MC4R in tilapia (70). We further confirmed the direct interaction and co-localization of both MC3R and MC4R with MRAP2 proteins on the plasma membrane by co-IP and BiFC assays (**Figure 7**). Our results were consistent with some previous studies in other vertebrates such as the clawed frog (45) and sea lamprey (54). Taken together, multiple studies from different species demonstrated that MRAP2 acted as a bi-functional player in modulating the MCRs trafficking to the plasma membrane, suggesting its species-specific role in modulating the cell surface expression of MC3R/MC4R.

The food deprivation experiment further confirmed the vital role of melanocortin signaling in regulating the appetite and energy homeostasis. Our results showed that axolotl *mc3r* and *agrp* significantly upregulated in the fasting hypothalamus, whereas the expression levels of axolotl *mrap2* and *pomc* significantly reduced in hypothalamus upon 3-week fasting (**Figure 8**). As a key regulator of central appetite, multiple studies had reported the significant expressional dynamics of *agrp* and *pomc* expression in the central nervous system upon fasting treatment. For example, the elevation of *Agrp* (81) and the inhibition of *Pomc* expression (82) were reported in mouse hypothalamus after fasting. Studies in teleost fish had shown consistent results, for instance, *agrp* was significantly upregulated whereas pomc was not altered by fasting in zebrafish adult brain (83). A significant hypothalamic upregulation of *agrp1* and downregulation of *pomca2* were identified after fasting in *Atlantic Salmon* (84).

In conclusion, our study comprehensively examined and elucidated the pharmacological and physiological regulation of central melanocortin signaling in the axolotl. We found a relatively conserved protein structure of axolotl MC3R, MC4R, and MRAP2 in comparison with other species, especially the TM regions. A co-expression profile of *mc3r*, *mc4r*, and *mrap2* along with *pomc* and *agrp* in the axolotl brain regions and the significant elevation of *mc3r* and *agrp* and reduction of *mrap2* and *pomc* in the hypothalamus upon fasting further confirmed the vital role of melanocortin signaling and their physiological interaction in regulating central feeding behavior and energy balance. The pharmacological profiles of axolotl MC3R and MC4R also demonstrated that both receptors could be activated by α-MSH and ACTH. The MRAP2 showed no effect on the cell surface expression level of MC3R, but significantly negative effect on the cell surface translocation of MC4R in a dose-dependent manner. The co-localization and functional complex formation of axolotl MC3R/MC4R with MRAP2 were confirmed by co-IP and BiFC assays *in vitro*. Taken together, pharmacological evaluation of the central melanocortin signaling of a primitive poikilotherm axolotl elucidated the vital physiological role in the regulation of appetite and energy balance in an amphibian species.

## Data Availability Statement

The datasets presented in this study can be found in online repositories. The names of the repository/repositories and accession number(s) can be found in the article/**Supplementary Material**.

## Ethics Statement

The animal study was reviewed and approved by the Tongji University.

## Author Contributions

XW, GL, JG, and ChZ participated in the design of the study. XW, SX, and XiL performed the experiments. LL, CoZ, WS, XL, YF, YL and HZ contributed to data collection and data analysis. XW, GL, and ChZ contributed to the manuscript writing. All authors contributed to the article and approved the submitted version.

## Funding

The work was supported by grants from the National Key Research and Development Program of China (Grant Nos. 2019YFA0111400 and 2017YFA0103902); the National Natural Science Foundation of China (Grant Nos. 31771283, 31771608, and 31801226); the Innovative Research Team of High-level Local Universities in Shanghai (Grant No. SSMU-ZDCX20180700) and a Key Laboratory Program of the Education Commission of Shanghai Municipality (Grant No. ZDSYS14005).

## Conflict of Interest

The authors declare that the research was conducted in the absence of any commercial or financial relationships that could be construed as a potential conflict of interest.

## Publisher’s Note

All claims expressed in this article are solely those of the authors and do not necessarily represent those of their affiliated organizations, or those of the publisher, the editors and the reviewers. Any product that may be evaluated in this article, or claim that may be made by its manufacturer, is not guaranteed or endorsed by the publisher.
