# Supplementary material for: Pharmacological Evaluation of Melanocortin 2 Receptor Accessory Protein 2 on Axolotl Neural Melanocortin Signaling
Source: Front Endocrinol (Lausanne). 2022 Feb 17;13:820896. doi: 10.3389/fendo.2022.820896 (PMC8891371; doi:10.3389/fendo.2022.820896)
Supplement: Supplementary file 1 [file Table_1.docx]

**Supplemental Table 1. Primers for cloning axolotl *mc3r*, *mc4r*, *mrap2*, *agrp* and *pomc* genes in this study.**

| **Usage** | **Gene name** | **Primer name** | **Sequences (5’ – 3’)** |
| --- | --- | --- | --- |
| **Full-length sequence identification** | *mc3r* | MC3R-F1 | GGGCATTCCAACTGCGCCTC |
|  |  | MC3R-R1 | AGCTCACATTGCTACACTATTCAATTTAA |
|  | *mc4r* | MC4R-F1 | CAGCAATTGAGTCCCACTCACACGG |
|  |  | MC4R-R1 | CTGCGAGAGCGTCTCTCCTCAGCAT |
|  | *mrap2* | MRAP2-F1 | ATGGTGGAGCTGAGAGATGCCGCACAC |
|  |  | MRAP2-R1 | CAAGGATGTGATGTAAGTTGTGGTG |
|  | *gapdh* | GAPDH-F1 | ATGAAAGTAGGAGTCAACGGATTTG |
|  |  | GAPDH-R1 | TTCCTTGCTGGCCATGTGGAC |
| **RT-PCR for tissue expression and qRT-PCR of fasting hypothalamic analysis** | *mc3r* | MC3R-F2 | TCTACGCTCTGCGCTATCAC |
|  |  | MC3R-R2 | TCACGTGCAGACGTGCAAAC |
|  | *mc4r* | MC4R-F2 | GTAGTTTAGCGGTCGCGGAT |
|  |  | MC4R-R2 | AGTACCTGTCCACCGCAATG |
|  | *mrap2* | MRAP2-F2 | GCAGAACCGTCAGAAAAGCTC |
|  |  | MRAP2-R2 | CCAGTTTGCAGTTTCTGGCT |
|  | *agrp* | AGRP-F2 | TTGCCGCTCAATCAGACGAA |
|  |  | AGRP-R2 | GCTCAAGTGGCAGTTATGGC |
|  | *pomc* | POMC-F2 | CAGCCACTGTCGGAGAACAT |
|  |  | POMC-R2 | GGAGTAGGAGCGCTTGTTGT |
|  | *gapdh* | GAPDH-F2 | GTTCCTGTCCCCAACGTCTC |
|  |  | GAPDH-R2 | ATCAGGTCCACAACACGCTG |
